# The Interplay between Osteoarthritis and the Microbiome-joint Axis: A Systemic Perspective on Novel Therapeutic Targets

**DOI:** 10.2174/0115733971450825260423042902

**Published:** 2026-04-24

**Authors:** Marco Valentini, Guido Maria Valentini, Ibrahim Akkawi, Sabrina Donati Zeppa, Alessia Bartolacci, Francesco Pegreffi, Hassan Zmerly

**Affiliations:** 1 Rheumatology Unit, San Pier Damiano Hospital, GVM, Faenza RA, Italy;; 2 Department of Biomedical Science, Humanitas University, Rozzano MI, Italy;; 3 Department of Orthopedics, Villa Erbosa Hospital, Bologna, Italy;; 4 Department of Human Science and Promotion of Quality of Life, San Raffaele Rome Open University, Rome, Italy;; 5 Department of Biomolecular Sciences, University of Urbino Carlo Bo, Urbino, 61029, Italy;; 6 Department of Medicine and Surgery, University of Enna Kore, Enna, Italy;; 7 Recovery and Functional Rehabilitation Unit, Umberto I Hospital, Enna, Italy;; 8 Department for Life Quality Studies, University of Bologna, Bologna, Italy

**Keywords:** Osteoarthritis, articular cartilage, gut microbiota, gut dysbiosis, low-grade inflammation, therapeutic targets

## Abstract

Osteoarthritis (OA) is a chronic and progressive joint disease involving the articular cartilage, synovium, subchondral bone, and ligaments, ultimately leading to pain, dysfunction, and, in advanced stages, joint destruction. Several factors contribute to the development and progression of OA, including genetic predisposition, biomechanical stress, metabolic imbalance, and chronic low-grade inflammation. Recently, a novel factor has emerged: the gut microbiome. Gut dysbiosis, defined as an alteration in gut microbiota homeostasis, can disrupt immune, metabolic, and inflammatory pathways, promoting systemic inflammation and accelerating degenerative changes in joint tissues. Conversely, restoration of a balanced gut microbiota may play a protective role and represent a promising avenue for innovative therapeutic strategies. The aim of this review is to analyse the relationship between gut dysbiosis and osteoarthritis, and to discuss potential therapeutic approaches targeting the microbiome to prevent disease progression.

## 1 INTRODUCTION

Osteoarthritis (OA) is among the leading causes of joint dysfunction worldwide and represents a major contributor to chronic disability [1-3]. Its prevalence translates into substantial social and economic burdens, including productivity losses and direct and indirect healthcare costs [1-5].

**Fig. (1):**
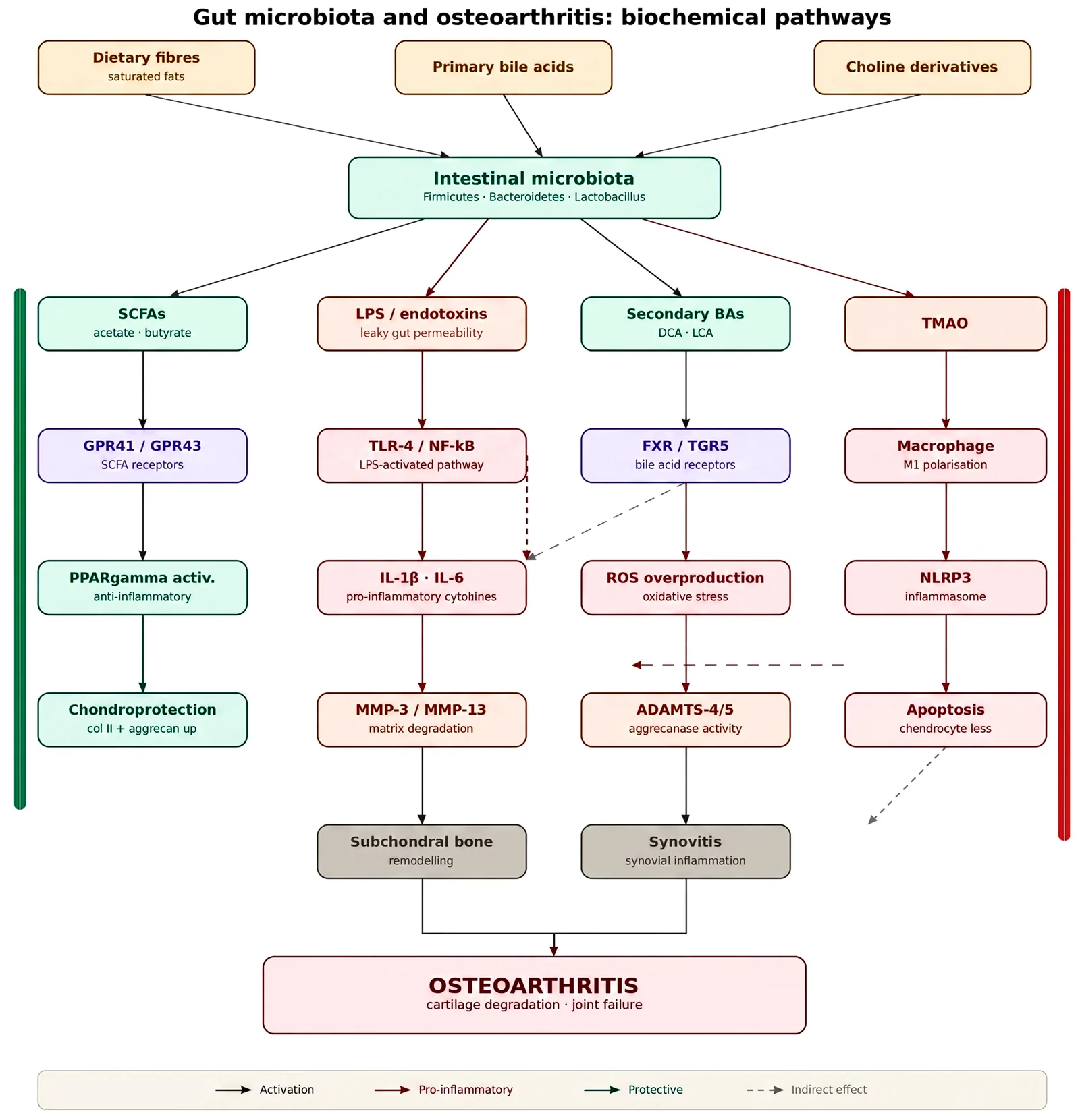
The biochemical pathway between the gut microbiome and osteoarthritis (OA); how alterations in gut microbiota can influence joint inflammation, cartilage degradation, and OA progression through metabolic, immune, and inflammatory mechanisms.

Traditionally, OA was regarded as a localized degenerative joint disease, in which the pathogenetic mechanism was primarily attributed to a disruption between subchondral bone and articular cartilage, ultimately leading to progressive cartilage degradation [5-7]. However, this reductionist view has been increasingly challenged. Current evidence highlights the multifactorial nature of OA pathogenesis (Fig. **1**), which involves not only mechanical and genetic factors, but also immuno-metabolic and systemic inflammatory processes [8-13]. Within this broader perspective, the gut microbiota has emerged as a key player [14-16].

This complex microbial ecosystem influences host physiology through immune, metabolic, and neuroendocrine pathways. Gut dysbiosis seems to be linked to altered immune responses and chronic inflammatory diseases such as cardiovascular disease, diabetes, obesity, neurodegeneration, and autoimmune disorders, as well as, more recently, osteoarthritis. Restoring gut microbiota balance could be a potential new therapeutic strategy in the management of early OA [17-24].

The purpose of this review is to examine the interplay between osteoarthritis and the gut microbiota-joint axis, emphasizing its systemic implications and exploring novel therapeutic targets aimed at modifying disease progression.

## 2 METHODS

This review was conducted through a structured search of the scientific literature across major biomedical databases, including PubMed/MEDLINE, Scopus, Web of Science, Embase, and the Cochrane Library. To ensure the broadest coverage, additional sources were explored through Google Scholar and grey literature. The search was carried out between January and April 2025 using a combination of Medical Subject Headings (MeSH) and free-text keywords such as “osteoarthritis”, “gut microbiota”, “gut microbiota-joint axis”, “gut dysbiosis”, “low-grade inflammation”, and “therapeutic targets.” Boolean operators (AND, OR) were applied to optimize the retrieval of relevant articles.

Studies on the "microbiome-joint axis" have become more extensive in recent years; therefore, works were included if they were published in English between 2000 and 2025, appeared in peer-reviewed journals, and investigated the interplay among the gut microbiota, systemic inflammation, and osteoarthritis.

Eligible publications encompassed experimental models, clinical trials, observational studies, systematic reviews, and meta-analyses. Non-English articles, conference abstracts, editorials, commentaries, and investigations unrelated to the microbiome-joint axis were excluded. The selection process involved an initial screening of titles and abstracts, followed by the full-text evaluation of potentially relevant articles. When duplicates were identified, the most complete version was retained.

Data from the included works were analysed with particular attention to the study design, methodology, and main findings.

The limitations of this study include the lack of protocol registration, noncompliance with PRISMA guidelines, and a lack of a formal assessment of study quality.

## 3 OA TREATMENT

Chronic low-grade inflammation represents a key factor underlying several degenerative diseases, including Osteoarthritis (OA) [8-13]. This persistent inflammatory state leads to structural alterations, including changes in the chondral matrix, fibrillation, and thinning of hyaline cartilage, subchondral bone sclerosis, and the subsequent formation of geodes and osteophytes, all of which contribute to progressive joint dysfunction [1, 2]. Chronic synovitis, frequently observed in OA patients, further exacerbates this degenerative cascade [11-13].

Conservative management of OA includes both non-pharmacological and pharmacological approaches [25-30]. Non-pharmacological strategies comprise weight reduction, physical exercise, and lifestyle modification [31-34]. Among pharmacological options, acetaminophen is the most frequently prescribed analgesic, although non-steroidal anti-inflammatory drugs (NSAIDs), cyclooxygenase-2 (COX-2) inhibitors, and opioids generally provide superior pain control [9, 27]. Importantly, prolonged courses of anti-inflammatory drugs, lasting approximately three months, are required to meaningfully impact the trajectory of joint damage and improve long-term outcomes [6, 7, 33, 34].

At the beginning of the 2000s, pharmaceutical research focused on developing selective COX-2 inhibitors to reduce adverse effects while maintaining efficacy. However, despite initial enthusiasm, these agents were later associated with long-term safety concerns, particularly cardiovascular risks, similarly to traditional NSAIDs [27].

Symptomatic slow-acting drugs for osteoarthritis (SYSADOAs), such as glucosamine and chondroitin sulphate, have been investigated for their potential to slow structural progression. Nonetheless, current evidence remains controversial as to whether they exert true disease-modifying effects or are limited to symptomatic relief [17, 28].

In cases of disease flare-ups, intra-articular injections, particularly corticosteroids and hyaluronic acid, represent valuable adjuncts for symptom management and the preservation of joint homeostasis [35].

Ongoing clinical trials are exploring the development of Disease-Modifying Osteoarthritis Drugs (DMOADs), targeting pathways such as Fibroblast Growth Factor (FGF), Wnt signalling, cathepsin K inhibition, interleukin-1 blockade, gene therapy, and bisphosphonates [36]. Despite these advances, real-world practice indicates that a substantial proportion of patients continue to experience cartilage damage progression, even with SYSADOAs and intra-articular treatments. This therapeutic gap highlights the need to explore novel systemic approaches [5, 37, 38].

The field of nanomedicine has opened transformative avenues for the management of OA, addressing the pharmacokinetic limitations of conventional anti-inflammatory drugs, such as poor bioavailability, non-specific biodistribution, and systemic toxicity. Nanoformulations-including polymeric nanoparticles, liposomes, solid lipid nanoparticles, dendrimers, and inorganic nanocarriers-enable targeted intra-articular delivery of therapeutic agents, prolonging drug retention within the synovial space and enhancing local bioavailability while minimizing systemic exposure. Among the most promising natural compound-based nanoformulations are those derived from terpenes, a diverse class of bioactive phytochemicals with well-documented anti-inflammatory, antioxidant, and chondroprotective properties [38].

Recently, the gut microbiota has emerged as a potential contributor to Osteoarthritis (OA) pathogenesis through its influence on immune, metabolic, and inflammatory pathways. Targeting dysbiosis and restoring microbiome homeostasis may therefore represent a promising frontier in the development of innovative treatments for OA [4, 18, 22].

## 4 OA AND GUT MICROBIOTA

Osteoarthritis (OA) is a chronic degenerative joint disease characterized by the progressive breakdown of articular cartilage, subchondral bone remodelling, and synovial inflammation, increasingly recognized as a condition with systemic metabolic underpinnings rather than a simple consequence of mechanical wear.

Recent studies show that low-grade chronic inflammation, which underlies many degenerative conditions like OA, may be linked to alterations in intestinal permeability and gut microbiota composition [39-41]. These systemic changes activate immune-inflammatory responses at both the intestinal and systemic levels, thereby contributing to the pathogenesis of osteoarthritis [42, 43].

Beyond OA, pathological alterations of the gut microbiota could influence chronic inflammatory rheumatic diseases, including rheumatoid arthritis, spondyloarthritis, and connective tissue disorders [44-48]. For example, several studies show how *Porphyromonas gingivalis* and *Prevotella* species could amplify the production of citrullinated antigens (CCP), a biomarker of rheumatoid arthritis severity. A growing body of evidence highlights significant pathophysiological overlap between OA and gout, both of which share mechanisms of crystal-induced or metabolite-driven synovial inflammation, oxidative stress, and immune dysregulation [45].

Several environmental and dietary factors like exposure to pesticides, food additives, nutritional disorders, antibiotic overuse, and reduction of immunoglobulin A (IgA) could alter gut microbiota, increase intestinal permeability, and disrupt tight junctions. These alterations facilitate the translocation of lipopolysaccharides (LPS) to antigen-presenting or submucosal dendritic cells, triggering immune activation and the release of pro-inflammatory cytokines [49-51]. Moreover, the translocation of bacterial products into the circulation, ultimately reaching the synovium, where they activate T cells and macrophages, fuelling joint inflammation [52, 53].

Targeting the gut microbiota through prebiotics, probiotics, postbiotics, and dietary interventions, therefore, represents a promising strategy to modulate systemic inflammation and slow OA progression. Preclinical studies further support the potential role of gut microbiota in OA progression. Huang *et al*. [49] and Collins *et al*. [50] reported a potential correlation between the elevated level of bacterial LPS and the acceleration of cartilage degeneration in animal models, whereas germ-free conditions seem to attenuate post-traumatic arthritis. In humans, Huang *et al*. observed, in dysbiotic individuals, an increase of LPS in the serum and synovial fluid, accompanied by synovial macrophage activation and worsening Western Ontario and McMaster Universities Arthritis Index (WOMAC) scores [49].

Experimental interventions modifying the microbiota also appear to influence OA outcomes. The administration of glucosamine, type II collagen, or chondroitin sulphate seems to be associated with favourable microbial shifts, including a reduction in pro-inflammatory *Proteobacteria*, lower systemic LPS levels, and increased short-chain fatty acids (SCFAs) and butyrate production [50]. These findings may explain the clinical improvements in joint pain and function reported with SYSADOAs [4, 17].

Among protective species, *Bifidobacteria* are notable for their ability to modulate innate immunity *via* Toll-like receptor 2 (TLR2) signalling [52-56]. Specific strains enhance dendritic cell maturation, promote interleukin-10 (IL-10) secretion, and inhibit TNF-α production, while also influencing host metabolic processes through vitamin activation and enzymatic pathways. These immunomodulatory properties highlight their potential to restore eubiosis and counteract OA-related inflammation.

A dietary imbalance may alter the *Firmicutes/Bacteroidetes* ratio and appears to be associated with systemic low-grade inflammation; diets rich in saturated fats seem to reduce the abundance of *Bacteroidetes* while promoting the growth of opportunistic species. These changes increase pathogen-associated molecular patterns (PAMPs) and damage-associated molecular patterns (DAMPs), which could perpetuate systemic inflammation and insulin resistance [17, 42]. In this context, Cani *et al*. [44] demonstrated that obesity and type II diabetes share a state of low-grade chronic inflammation driven by alterations of gut microbiota, and other studies have seen this correlation in individuals with metabolic syndrome [8, 42].

Clinical data further support this link. Favero *et al*. [52] demonstrated that patients with erosive hand OA exhibited elevated serum myeloperoxidase, a marker of leucocyte activity, along with higher visfatin levels, particularly in individuals with diabetes mellitus. Conversely, microbiota-derived metabolites such as short-chain fatty acids (SCFAs), notably butyrate, exert a potential anti-inflammatory effect. SCFAs enhance insulin sensitivity, reinforce intestinal barrier integrity, and modulate immune homeostasis by promoting regulatory T cell differentiation *via* histone H3 acetylation, which silences the pro-inflammatory Foxp3 gene [40].

Collectively, these findings reinforce the hypothesis that alterations of gut microbiota may contribute to systemic immunophlogosis and influence OA progression, opening the way to new potential microbiota-targeted therapeutic strategies [53-59].

## 5 NEW THERAPEUTIC TARGETS: PROBIOTICS AND MICROBIOME-DIRECTED STRATEGIES

Osteoarthritis (OA) may be considered a condition at the crossroads between immune-mediated and dysmetabolic inflammation. In this context, modulation of the gut microbiome has emerged as a potential therapeutic avenue. Probiotics, defined as live or inactivated microorganisms that, when administered in adequate amounts, beneficially influence host health, have been investigated for their potential to modify OA progression by restoring eubiosis and attenuating low-grade systemic inflammation [58, 59].

Preclinical work by Henrotin *et al*. explored the effects of a Lyophilized Inactivated Culture (LIC) of *Bifidobacterium longum* CBi0703 in the Dunkin Hartley guinea pig model of spontaneous OA [58]. The treatment groups received either CBi0703 alone or in combination with vitamin C, with outcomes assessed through histological scoring (Osteoarthritis Research Society International [OARSI] scores) and biochemical markers including Coll2-1, PIIANP, Fib3-2, and osteocalcin. The study showed no significant difference in the global OARSI score; however, improvement in the cartilage structure sub-score was noted in the group receiving CBi0703 plus vitamin C. Furthermore, a reduction in the collagen type II degradation marker (Coll2-1) was observed in the CBi0703 group, while the combination treatment increased PIIANP, a marker of type II collagen synthesis. Although mild synovial hyperplasia persisted in all groups, these findings suggest a chondroprotective effect of CBi0703, particularly in combination with vitamin C. A limitation of this investigation was the absence of a vitamin C-only control arm [58].

On the clinical side, a randomized, double-blind, placebo-controlled trial involving 537 patients with knee OA investigated the effects of *Lactobacillus casei* Shirota (LcS) administered for six months [59]. Patients receiving LcS demonstrated a statistically significant improvement in pain and function, as assessed by the WOMAC index and the visual analogue scale (VAS), along with a reduction in high-sensitivity C-reactive protein (hs-CRP) compared with placebo. These results provide evidence that probiotics could reduce OA symptoms by modulating systemic inflammation, supporting their potential as an adjunctive therapy in knee OA [59].

Additional preclinical evidence comes from the administration of *Clostridium butyricum* GKB7 in an anterior cruciate ligament transection (ACLT) rat model of OA. Oral treatment with this strain alleviated pain-related behaviours, as well as reducing osteochondral damage (as measured by OARSI scoring) and downregulating the expression of IL-1β and TNF-α in both cartilage and synovium. These findings suggest that *C. butyricum* may represent a novel disease-modifying candidate in OA management [60]. Recent studies further support this concept, concluding that both preclinical and clinical studies indicate probiotics can alleviate OA-related pain and potentially slow disease progression by positively modulating the gut microbiota and reducing chronic low-grade inflammation [60-64].

Patent literature also reflects a growing interest in microbiome-based strategies for OA and joint pain, underscoring the translational potential of this therapeutic approach [14, 65-69].

It is important to consider that the effects of probiotics are strain-specific. There are several limitations in most clinical studies on probiotics in the literature, including heterogeneity in probiotic strains, dosage regimens, and treatment duration, as well as structural outcomes. Based on these limitations, more structured randomized clinical trials are needed to address these issues and achieve consistent and reliable results.

## CONCLUSION

The intestinal microbiota, in addition to being a central component of mucosal immunity, represents a key interface of the human body’s systemic nature, mediating complex immuno-metabolic interactions. Increasing evidence suggests that microbiome dysregulation contributes to chronic low-grade inflammation, a shared mechanism underlying numerous degenerative conditions, including osteoarthritis (OA). Such insights expand the understanding of OA beyond a purely local joint disorder and position it within a systemic and multifactorial framework.

Although current evidence is still preliminary, available data support the hypothesis that restoring eubiosis may beneficially influence the long-term course of OA, particularly in patients with dysmetabolic phenotypes. Accordingly, evaluation of the gut microbiome should be integrated into the clinical assessment of OA, alongside conventional diagnostic and therapeutic strategies. This comprehensive perspective acknowledges OA as a heterogeneous disease, in which each causal or contributory factor, whether biomechanical, genetic, metabolic, inflammatory, or microbial, must be identified and addressed with specific, targeted interventions.

This review underscores the need for a 360-degree approach to OA, one that not only alleviates symptoms but also confronts the systemic drivers of disease progression. By extending awareness of the microbiome-joint axis, it highlights new opportunities for innovation in prevention and treatment. Ultimately, the future of OA management will rely on integrated, personalized strategies that combine conventional therapies with microbiome-targeted interventions, opening a new frontier in musculoskeletal medicine where joints, metabolism, and microbiota are viewed as inseparable partners in health and disease.

## LIST OF ABBREVIATIONS

COX-2: Cyclooxygenase-2
DMOADs: Disease-modifying Osteoarthritis Drugs
FGF: Fibroblast Growth Factor
LcS: Lactobacillus casei Shirota
LIC: Lyophilized Inactivated Culture
LPS: Lipopolysaccharides
NSAIDs: Non-steroidal Anti-inflammatory Drugs
OA: Osteoarthritis
OARSI: Osteoarthritis Research Society International
SCFAs: Short-chain Fatty Acids
SYSADOAs: Symptomatic Slow-acting Drugs for Osteoarthritis
TLR2: Toll-like Receptor 2
WOMAC: Western Ontario and McMaster Universities Arthritis Index
